# Impact of ventilator-associated pneumonia on mortality and epidemiological features of patients with secondary peritonitis

**DOI:** 10.1186/s13613-016-0137-5

**Published:** 2016-04-18

**Authors:** María Heredia-Rodríguez, María Teresa Peláez, Inmaculada Fierro, Esther Gómez-Sánchez, Estefanía Gómez-Pesquera, Mario Lorenzo, F. Javier Álvarez-González, Juan Bustamante-Munguira, José María Eiros, Jesús F. Bermejo-Martin, José I. Gómez-Herreras, Eduardo Tamayo

**Affiliations:** Anaesthesiology and Surgical Critical Care Department, Hospital Clínico Universitario de Valladolid, Avenida Ramón y Cajal, 3, 47005 Valladolid, Spain; Group of Biomedical Research in Critical Care Medicine (BioCritic), Hospital Clínico Universitario de Valladolid, Valladolid, Spain; Department of Pharmacology and Therapeutics, Faculty of Medicine, University of Valladolid, Valladolid, Spain; Department of Cardiovascular Surgery, Hospital Universitario de La Princesa, Madrid, Spain; Department of Microbiology, Faculty of Medicine, University of Valladolid, Valladolid, Spain; Investigación Médica en Infección e Inmunidad (IMI), Hospital Clínico Universitario de Valladolid-IECSCYL, Valladolid, Spain

## Abstract

**Background:**

Despite the significant impact of nosocomial infections on the morbidity and mortality of patients staying in the intensive care unit (ICU), no study over the past 20 years has focused specifically on VAP following secondary peritonitis. The objective of the present study was to determine in-hospital mortality and epidemiological features attributed to ventilator-associated pneumonia (VAP) following secondary peritonitis.

**Methods:**

Prospective observational study involved 418 consecutive patients admitted in the ICU. Univariate and multivariate analyses were performed to identify risk factors associated with mortality and development of VAP.

**Results:**

The incidence of VAP following secondary peritonitis was 9.6 %. Risk factors associated with the development of VAP were hospital-acquired peritonitis, requiring >48 h of mechanical ventilation, and SOFA score. The onset of VAP was late in majority of patients. VAP was developed about 16.8 days after the initiation of the peritonitis. Etiological microorganisms responsible for the peritonitis were different than for VAP. The 90-day in-hospital mortality rate was 47.5 % of VAP patients. Independent factors associated with 30- to 90-day in-hospital mortality were VAP and SOFA.

**Conclusions:**

In light of the impact on morbidity and mortality in the ICU, more attention should be given to the concurrent features among VAP and secondary peritonitis.

## Background

Intra-abdominal infections (IAIs) are one of the most important causes of mortality in the intensive care unit (ICU) [1]. Secondary peritonitis constitutes 80–90 % of cases of IAIs and is originated from the microbiological infection of the gastrointestinal tract by the perforation of hollow organs, ischemia, malignancy, and perioperative complications (anastomotic leakage, intraoperative contamination) [2, 3]. Secondary peritonitis can be classified in community-acquired and hospital-acquired, this latter associated with microorganisms presenting antibiotic resistance [2]. Mortality rate due to secondary peritonitis ranges approximately between 10 and 20 % [4–6]. During the management, the clinical outcome of the patient may be critically compromised by the development of nosocomial infections [7]. Ventilator-associated pneumonia (VAP) is a type of hospital-acquired pneumonia that is developed after at least 48 h of the patient’s intubation [8]. VAP is the most frequent of the nosocomial infections occurring in the ICU, affecting to 9–27 % of all intubated patients [9]. The VAP is associated with an increased length of hospital stay, of about 4–13 days, and hospital costs [10–13]. In our knowledge, there are only in the literature three studies analyzing specifically clinical and epidemiological aspects of the development of VAP following secondary peritonitis. The first one was a retrospective study of 1982, which reported clinical outcomes of 143 patients with intra-abdominal abscesses, and revealing an incidence of VAP of 28.7 % of the patients, and a mortality rate attributed to VAP of 65.9 % [14]. The second one was a prospective study published in 1991 comparing clinical outcomes between nosocomial pneumonia and recurrent IAI [15]. The incidence of VAP was 19.7 % of cases, and the mortality rates were 53 % for the group of patients with pneumonia and no recurrent IAI, and 75 % of those with both conditions. Finally, the third study, of 2006, included retrospectively medical records from 618,495 patients undergoing intra-abdominal surgery [16]. From them, 13,292 patients developed subsequently pneumonia, and the mortality rate was of 10.7 %.

Although there are extensive studies analyzing secondary IAIs or VAP in the ICU, in our knowledge, studies focusing specifically on VAP following secondary peritonitis are scarce and date mainly from two decades ago. Furthermore, there are some issues that remain being characterized, such as the lapse time between the starting of the peritonitis and VAP onset, and whether or not the etiologic agents responsible for IAIs are the same that for VAP, which is critical for the selection of the empirical antibiotic therapy. Early VAP onset has been associated with better prognosis, while the late one has the highest mortality rates and is often associated with multidrug-resistant microorganisms [8]. Our working hypothesis is that pneumonia increases the mortality in patients developing peritonitis. Therefore, the objective of the study was to determine in-hospital mortality and epidemiological features attributed to VAP following secondary peritonitis.

## Methods

This prospective observational study involved consecutive patients admitted in the ICU of the clinical university hospital of Valladolid between May 2008 and May 2015 for the management of a secondary peritonitis. All patients, or family members, signed the written consent form to participate in the study. The collection of respiratory and blood samples, for microbiological examinations, was required for the inclusion in the study. Patients presenting primary peritonitis or those who refused to sign the consent form were excluded from the study. One of the investigators made daily rounds in the ICU to identify eligible patients and determine the onset of VAP. Because of the observational nature of the study, investigators did not interact with ICU treating physicians for the diagnosis or management of VAP. To test our working hypothesis, the primary endpoint was to evaluate whether or not VAP patients had a higher mortality rate than non-VAP patients. Secondary endpoints included the identification of variables potentially associated with in-hospital mortality and with the development of VAP. Procedures were performed in accordance with guidelines established by the hospital’s ethics committee and the Declaration of Helsinki.

### Surgical procedures and microbiological management

The surgery was performed by an experienced and trained team following the guidelines for the treatment of complicated IAIs [17]. A laparoscopy or laparotomy was performed taking into account the diagnosis and the preference of the surgeon. Peritoneal fluid was sampled to detect microbiological and mycological activity. The empirical antimicrobial therapy was started as soon as possible and consisted in the administration of amoxicillin/clavulanic acid or meropenem plus linezolid if community-acquired or hospital-acquired peritonitis, respectively. Treatment against yeast infection was only considered in the case of organ failure. Ranitidine (50 mg intravenously every 12 h) was administered for gastric protection within the first 24 h of admission in the ICU. Mouthwashes with chlorhexidine were carried out twice a day [18]. The adequacy of source control was confirmed by specialists in the ICU. The empirical antibiotic treatment for VAP was based on identifying the most common pathogens associated with VAP in the ICU, following international guidelines, including the initial empirical treatment of methicillin-resistant S. aureus with linezolid or teicoplanin and of *P. aeruginosa* with at least one of the following antibiotics: imipenem, cefepime, or piperacillin–tazobactam, in association with amikacin or ciprofloxacin [19].

### Diagnosis of VAP

According to the definition of the Centers for Disease Control and Prevention, VAP was diagnosed upon the presence of new and/or progressive pulmonary infiltrates on a chest radiograph plus 2 or more of the following criteria: fever (≥38.5 °C) or hypothermia (<36 °C), leukocytosis (≥12 × 10^9^/L), positive pleural fluid culture, purulent tracheobronchial secretions, or a reduction in PaO_2_/FIO_2_ of at least 15 % in the previous 48 h, a cavitating infiltrate, and/or evidence of bronchiolitis, neutrophilic alveolitis, and consolidation [20, 21]. The diagnosis also included those patients with a Pugin score greater than 6 [22]. The confirmation of the diagnosis included the isolation of at least one pathogenic microorganism in significant bacterial counts, i.e., ≥10^3^ colony-forming units (CFU)/mL for protected specimen brush, ≥10^4^ CFU/mL in case of bronchoalveolar lavage, and ≥10^5^ CFU/mL for endotracheal aspiration. These cutoffs were not modified in patients receiving antimicrobial therapy at the time of VAP diagnosis. Coagulase-negative *Staphylococcus*, *Corynebacterium* spp., *Candida* spp., *Viridans* group *streptococci*, and *Neisseria* spp. were no considered pathogenic microorganisms. Special attention was given to species isolated from both peritoneal fluid and lungs.

### Outcome variables and statistical analysis

In-hospital mortality (at 30 days, 30–90 days, and 90 days) was differentiated from caused by the severity of the peritonitis or intraoperative and postoperative events. Patients were evaluated for VAP during mechanical ventilation and within 48 h after extubation. Hospital-acquired infection was defined when occurred ≥48 h after admission. Early or late VAP onset was established depending on whether VAP was developed before or later the 4th day since the initiation of the peritonitis and the mechanical ventilation. Regarding the results of the antibiogram, the treatment was classified as adequate or inadequate. Multidrug resistance was considered when species showed resistance to at least three groups of antibiotics. Categorical variables were expressed as absolute and relative (%) frequencies, whereas continuous ones as the median and the standard deviation (SD) or the median and the interquartile range (IQR). Differences between groups were compared by using the *t* test, with continuous variables, and by Chi-square test or Fisher’s exact test, with categorical ones. A univariate analysis, classifying patients in survivors and nonsurvivors, was also carried out to identify potential demographic and clinical factors associated with in-hospital mortality. Kaplan–Meier analysis was performed to compare overall survival regarding the development of VAP. Stepwise logistic regression analyses were performed to identify factors associated with in-hospital mortality and with the development of VAP (odds ratio, OR, and 95 % confidence interval, 95 % CI). Independent variables introduced in the models were carefully selected to avoid confounding effects. The statistical significance was established for *p* ≤ 0.05. All statistical procedures were performed with SPSS 19.0 software.

## Results

### Clinical and microbiological characteristics of patients

From a total of 418 patients presenting secondary peritonitis, 40 subsequently did develop VAP (9.6 %) and 378 did not (90.4 %; Table 1). The mean lapse time between the starting of the secondary peritonitis and the development of VAP was 16.8 ± 15.1 days (community-acquired 14.6 ± 14.5 days and hospital-acquired 21.8 ± 15.7 days). The VAP onset was early in 12 patients (30.0 %) and late in 28 (70.0 %; *p* < 0.001). The mean age of patients was 71.1 ± 11.0 years for those with VAP and 70.0 ± 13.3 years for those without VAP. Septic shock was higher in VAP patients (82.5 %) than non-VAP (61.4 %), whereas severe sepsis was opposite, 17.5 versus 38.6 % of patients, respectively. The infection was mainly hospital-acquired (70.0 %) in VAP patients, whereas community-acquired (52.1 %) in non-VAP. The main cause of peritonitis was bowel perforation in both groups (47.5 vs 43.6 %, respectively). Colon/rectum (50.0 vs 40.7 %) and small bowel (17.5 vs 19.0 %) were the most frequent locations of the peritonitis. The acute physiology and chronic health evaluation II (APACHE II) score and the Sepsis-Related Organ Failure Assessment (SOFA) score were significantly higher (*p* = 0.007 and *p* < 0.001) in VAP patients (15.95 ± 4.29 and 8.10 ± 2.50) than in non-VAP (13.65 ± 5.16 and 6.22 ± 2.46, respectively). A significantly (*p* < 0.001) higher number of VAP patients (62.5 %) received low-dose steroid therapy than non-VAP patients (25.7 %). VAP patients required significantly (*p* < 0.001) more days of mechanical ventilation (8.91 ± 14.49 days) than non-VAP (2.61 ± 6.19 days). More than 48 h of mechanical ventilation was required in a higher number of VAP patients (47.5 %) than in non-VAP (20.4 %; *p* < 0.001). The stay in the ICU and the hospital were significantly longer (*p* < 0.001) in VAP patients (median 9.0 days; IQR 7.0–30.0 days; and median 45.0 days; IQR 29.0–61.0 days, respectively) than in non-VAP (median 3.0 days; IQR 1.0–7.0 days; and median 20.0 days; IQR 11.0–34.0 days, respectively; Table 1). The most frequent species isolated from lungs of VAP patients were *Acinetobacter spp*. (45.0 % of patients), *Klebsiella spp*. (17.5 %), and *P. aeruginosa* (17.5 %) and from their peritoneal fluid were *Enterococcus spp*. (37.5 %), *E. coli* (35.0 %), *Klebsiella spp*. (25.0 %), and anaerobes (25.0 %; Table 2). All microorganisms given in Table 2 are associated with VAP. In lungs from non-VAP patients, the main species isolated were *anaerobes* (0.3 % of patients) and from their peritoneal fluid were *E. coli* (28.6 %), anaerobes (27.5 %), and *Enterococcus spp*. (24.1 %). Only three patients presented the same species in the peritoneal fluid and in lungs (two patients with *Klebsiella spp*. and one with *P. aeruginosa*; *p* < 0.001). VAP had a polymicrobial origin in four patients. None of the patients showed more than one VAP episode. Multidrug-resistant species were isolated from 25 VAP patients (62.5 %; *p* = 0.02). Regarding the antibiogram, the antibiotic treatment was therefore adequate in 22 VAP patients (55.0 %; *p* = 0.37).

**Table 1:** Demographic and clinical characteristics 24 h after the admission in the ICU in patients presenting secondary peritonitis regarding the subsequent development of ventilator-associated pneumonia

	VAP patients (*n* = 40)	Non-VAP patients (*n* = 378)	*P* value
Age (mean years ± SD)	71.1 ± 11.0	70.0 ± 13.3	0.61
Sex male [*n* (%)]	31 (77.5)	217 (57.4)	0.014
Comorbidities [*n* (%)]			
*Diabetes mellitus*	33 (82.5)	283 (74.3)	0.02
Hypertension	21 (52.5)	194 (51.3)	0.81
Malignant neoplasm	16 (40.0)	168 (44.4)	0.63
Obesity	5 (12.5)	52 (13.8)	0.83
Chronic renal failure	5 (12.5)	34 (9.0)	0.46
Immunosuppression	1 (2.5)	13 (3.4)	
Liver disease	2 (5.0)	11 (2.9)	0.46
Acute renal failure, dialysis	2 (5.0)	7 (1.9)	
Postoperative status [*n* (%)]			0.001
Septic shock	33 (82.5)	232 (61.4)	
Severe sepsis	7 (17.5)	146 (38.6)	
Type of infection [*n* (%)]			0.007
Community-acquired	12 (30.0)	181 (47.9)	
Hospital-acquired	28 (70.0)	197 (52.1)	
Etiology of peritonitis [*n* (%)]			0.72
Bowel perforation	19 (47.5)	165 (43.6)	
Anastomotic leakage	6 (15.0)	74 (19.6)	
Biliary pathology	6 (15.0)	44 (11.6)	
Ischemia	3 (7.5)	34 (9.0)	
Abdominal Abscess	2 (5.0)	36 (9.5)	
Pancreatitis	4 (10.0)	18 (4.8)	
Bladder perforation	0 (0.0)	3 (0.8)	
Uterine perforation	0 (0.0)	3 (0.8)	
Vesical perforation	0 (0.0)	4 (1.1)	
Location of the peritonitis^†^ [*n* (%)]			0.75
Colon/rectum	20 (50.0)	154 (40.7)	
Small bowel	7 (17.5)	72 (19.0)	
Biliary pathology	5 (12.5)	60 (15.9)	
Stomach and duodenum	1 (2.5)	31 (8.2)	
Pancreas	4 (10.0)	23 (6.1)	
Appendix	2 (5.0)	16 (4.2)	
Bladder	0 (0.0)	10 (2.6)	
Various	1 (2.5)	8 (2.1)	
Uterus/fallopian tubes	0 (0.0)	4 (1.1)	
Clinical score			
APACHE II (mean ± SD)	15.95 ± 4.29	13.65 ± 5.16	0.007
SOFA (mean ± SD)	8.10 ± 2.50	6.22 ± 2.46	<0.001
Postoperative management			
Low-dose steroid therapy [*n* (%)]	25 (62.5)	97 (25.7)	<0.001
Blood transfusions, units	3.50 ± 7.44	2.37 ± 4.89	0.192
Politransfusion (>10 units) [*n* (%)]	6 (15.0)	45 (11.9)	0.57
Mechanical ventilation			
Total duration (days ± SD)	8.91 ± 14.49	2.61 ± 6.19	<0.001
Patients requiring >48 h [*n* (%)]	19 (47.5)	77 (20.4)	<0.001
Time for VAP onset (mean days ± SD)	16.8 ± 15.1	–	
Clinical outcome			
Stay at ICU, median days (IQR)	9.0 (7.0–30.0)	3.0 (1.0–7.0)	<0.001
Total stay at the hospital, median days (IQR)	45.0 (29.0–61.0)	20.0 (11.0–34.0)	<0.001
Mortality after 30 days [*n* (%)]	7 (17.5)	76 (20.1)	0.69
Mortality after 90 days [*n* (%)]	18 (47.5)	96 (25.4)	0.008

*VAP* ventilator-associated pneumonia, *SD* standard deviation, *APACHE II* Acute Physiology and Chronic Health Evaluation II, *SOFA* Sepsis-Related Organ Failure Assessment, *ICU* intensive care unit, *IQR* interquartile range

^†^In some patients, the infection extended into more than one location

**Table 2:** Microorganisms isolated from lungs and peritoneal fluid associated with VAP in patients with secondary peritonitis

	Lungs		Peritoneal fluid	
	VAP patients (*n* = 40)	Non-VAP patients (*n* = 378)	VAP patients (*n* = 40)	Non-VAP patients (*n* = 378)
Gram-positive cocci				
Methicillin susceptible *Staphylococcus aureus*	6 (15.0)	0 (0.0)	1 (2.5)	8 (2.1)
Methicillin-resistant *Staphylococcus aureus*	2 (5.0)	0 (0.0)	0 (0.0)	2 (0.5)
*Staphylococcus epidermidis*	0 (0.0)	0 (0.0)	7 (17.5)	26 (6.9)
Other *Staphylococcus* spp.	2 (5.0)	0 (0.0)	0 (0.0)	16 (4.2)
*Streptococcus* spp.	0 (0.0)	0 (0.0)	4 (10.0)	33 (8.7)
*Enterococcus* spp.	1 (2.5)	0 (0.0)	15 (37.5)	91 (24.1)
Other	0 (0.0)	0 (0.0)	4 (10.0)	10 (2.6)
Gram-negative bacilli				
*Klebsiella* spp.	7 (17.5)	0 (0.0)	10 (25.0)	27 (7.1)
*Enterobacter* spp.	1 (2.5)	0 (0.0)	6 (15.0)	25 (6.6)
*Escherichia coli*	4 (10.0)	0 (0.0)	14 (35.0)	108 (28.6)
*Pseudomonas aeruginosa*	7 (17.5)	0 (0.0)	6 (15.0)	3 (0.8)
*Acinetobacter* spp.	18 (45.0)	1 (0.3)	2 (5.0)	5 (1.3)
Other *Enterobacteriaceae*	2 (5.0)	0 (0.0)	0 (0.0)	8 (2.1)
Anaerobes	1 (2.5)	1 (0.3)	10 (25.0)	104 (27.5)

Percentages may sum more than 100 % because more than one pathogen could have been found in an individual patient

### Relationship between secondary peritonitis, pneumonia, and mortality

**Fig. 1:**
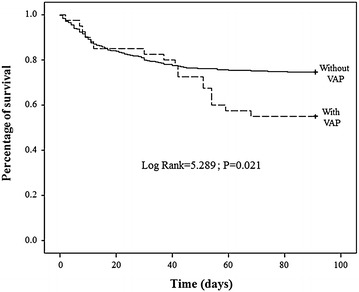
Kaplan–Meier analysis showing the percentage of survival between patients with and without ventilator-associated pneumonia

Mortality at 30 days was not different between groups and however at 90 days was significantly higher (*p* = 0.008) in VAP patients (45.0 %) than in non-VAP (5.8 %). Kaplan–Meier survival analysis revealed that the percentage of survival was different between VAP and non-VAP patients (log rank = 5.289; *p* = 0.021; Fig. 1), indicating higher values for non-VAP patients. Both survival curves diverged after the day 40th of admission in the ICU.

### Factors associated with in-hospital mortality and development of VAP

By classifying patients in survivors (*n* = 304, 72.7 %) and nonsurvivors (*n* = 114, 27.3 %), the univariate analysis demonstrated that in-hospital mortality was significantly associated with 19 demographic or clinical variables (Table 3). The logistic regression model indicated that independent factors associated with 30-day in-hospital mortality were age (OR 1.038; CI 95 % 0.003–1.013; *p* = 0.003), SOFA (OR 1.329, CI 95 % 0.0001–1.171; *p* < 0.001), and severe sepsis/septic shock (OR 3.105; CI 95 % 0.013–1.271; *p* = 0.013). Stepwise logistic regression model to identify independent factors associated with in-hospital mortality at 30, 30–90, and 90 days in patients with secondary peritonitis is given in Table 4. Independent factors associated with 30- to 90-day in-hospital mortality were SOFA (OR 1.373, CI 95 % 0.0001–1.151; *p* < 0.001), and VAP (OR 3.777, CI 95 % 0.006–1.475; *p* = 0.006). Factors associated with 90-day in-hospital mortality were age (OR 1.036; CI 95 % 0.002–1.013; *p* = 0.002), SOFA (OR, 1.247, CI 95 % 0.006–1.065; *p* = 0.006), creatinine (OR 1.351; CI 95 % 0.042–1.011; *p* = 0.042), and severe sepsis/septic shock (OR 2.967; CI 95 % 0.004–1.402; *p* = 0.004). Finally, independent factors associated with the development of VAP were hospital-acquired peritonitis (OR 2.873; CI 95 % 1.299–6.369; *p* = 0.009), SOFA (OR 1.325; CI 95 % 1.126–1.559; *p* = 0.001), and requiring >48 h of mechanical ventilation (OR 2.359; CI 95 % 1.074–5.181; *p* = 0.032).

**Table 3:** Significant demographic and clinical variables potentially associated with in-hospital mortality

	Nonsurvivors (*n* = 114)	Survivors (*n* = 304)	*P* value
Age (mean years ± SD)	74.69 ± 10.43	68.38 ± 13.58	0.006
Comorbidities [*n* (%)]			
Chronic renal failure	21 (18.4)	18 (5.9)	<0.001
Acute renal failure, dialysis	3 (2.6)	6 (2.0)	<0.001
Immunosuppression	11 (9.6)	4 (1.3)	<0.001
Postoperative status			0.001
Severe sepsis	12 (10.5)	139 (45.7)	
Septic shock	100 (87.7)	163 (53.6)	
Biochemical parameters at ICU (mean ± SD)			
Sodium (mEq/L)	137.64 ± 6.8	136.05 ± 4.81	<0.001
Creatinine (mg/dL)	2.18 ± 1.71	1.21 ± 0.77	<0.001
Lactate (mmol/L)	34.90 ± 26.16	24.13 ± 19.28	0.005
Procalcitonin (ng/mL)	24.48 ± 36.42	16.27 ± 30.28	0.043
HCO_3_^−^ (mEq/L)	20.42 ± 7.28	21.73 ± 5.52	0.030
Postoperative management			
Low-dose steroid therapy	25 (21.9)	97 (31.9)	<0.001
Blood transfusions, units	4.43 ± 6.90	1.74 ± 4.16	<0.001
Politransfusion (>10 units) [*n* (%)]	26 (22.8)	25 (8.2)	<0.001
Mechanical ventilation			
Total duration (days ± SD)	7.38 ± 11.70	1.69 ± 4.48	<0.001
Patients requiring >48 h [*n* (%)]	50 (43.9)	46 (15.1)	<0.001
Clinical outcome			
Stay at ICU, median days (IQR)	8.0 (3.8–14.3)	3.0 (1.0–6.0)	<0.001
Total stay at the hospital, median days (IQR)	21.0 (9.3–43.8)	21.0 (12.3–35.0)	0.004
VAP	18 (15.8)	22 (7.2)	0.008

*SD* standard deviation, *ICU* intensive care unit, HCO_3_^−^ bicarbonate, *VAP* ventilator-associated pneumonia, *IQR* interquartile range

**Table 4:** Logistic regression models to identify factors associated with in-hospital mortality and with the development of VAP

	OR	95 % CI	*P* value
In-hospital mortality			
30-day in-hospital mortality			
Age (years)	1.038	1.013–1.064	0.003
SOFA score	1.329	1.171–1.510	<0.001
Severe sepsis/septic shock	3.105	1.271–7.588	0.013
30- to 90-day in-hospital mortality			
SOFA score	1.373	1.151–1.637	<0.001
VAP	3.777	1.475–9.671	0.006
90-day in-hospital mortality			
Age (years)	1.036	1.013–1.060	0.002
SOFA score	1.247	1.065–1.461	0.006
Creatinine (mg/dL)	1.351	1.011–1.805	0.042
Severe sepsis/septic shock	2.967	1.402–6.278	0.004
Development of VAP			
Hospital-acquired peritonitis	2.873	1.299–6.369	0.009
SOFA score	1.325	1.126–1.559	0.001
Requiring >48 h of mechanical ventilation	2.359	1.074–5.181	0.032

*OR* odds ratio, *CI* confidence interval, *SOFA* Sepsis-Related Organ Failure Assessment, *VAP* ventilator-associated pneumonia

## Discussion

Despite the significant impact of nosocomial infections on morbidity and mortality of patients staying in the ICU, no study over the last 20 years has determined the clinical, epidemiological, and microbiological features of VAP following secondary peritonitis. Therefore, the goal of the present study was to complete and update such lacking information. The most relevant results from our study included: (1) an updated incidence value of VAP of 9.6 %; (2) risk factors associated with the development of VAP including hospital-acquired peritonitis, requiring >48 h of mechanical ventilation, and SOFA score; (3) mainly late onset of VAP, and caused by multidrug-resistant microorganisms intrinsically different for each condition; (4) the 90-day in-hospital mortality rate of 47.5 % of VAP patients; and (5) independent factors associated with 30- to 90-day in-hospital mortality including VAP and SOFA score.

Overall incidence of VAP reported in our study was 9.6 % of patients who underwent surgery due to peritonitis, a value significantly lower than previous studies, ranging between 20 and 30 % [14, 15]. A possible explanation may derive from the fact that, in our study, VAP included both hospital- and community-acquired cases; however, previous studies only included hospital-acquired cases, which are associated with higher incidence rates. Moreover, the reduction in the impact of VAP over the years may be a result of the implementation of effective preventive strategies in the ICU, such as the Spanish national VAP prevention bundle called “zero VAP,” based on good general practices for control of the infection and pathogenic-tailored strategies [23, 24]. The International Nosocomial Infection Control Consortium reported a decrease in incidence from 15 to 8 % in a surveillance study conducted from 2004 to 2009 [25, 26].

There are many factors potentially associated with the development of VAP, including preexisting medical conditions in the patient (such as immunosuppression or chronic obstructive lung disease), body position, level of consciousness, nasotracheal intubation, duration of the mechanical ventilation, ventilator circuit-related factors, enteral nutrition, or personnel-related factors (such as inadequate hand hygiene or change in gloves between patients) [12, 27, 28]. In our study, hospital-acquired peritonitis, requiring >48 h of mechanical ventilation, and SOFA score were independent factors for VAP development. The duration of ventilation has been positively correlated with the development of VAP, although this potential risk seems not to be constant over the time [12]. Specifically, the risk of VAP has been estimated in 3 % per day during the first week of mechanical ventilation, 2 % in the second week, and 1 % in the subsequent weeks [27]. A high SOFA score at admission in the ICU has been associated with mortality in VAP patients [29]; for this reason, a high score in multiple organ dysfunction, concomitant with a probably immunosuppression status and/or other underlying medical conditions, may be a cause for the development of such opportunistic infection. Similarly, hospital-acquired peritonitis is associated with microorganisms presenting antibiotic resistance, poor outcomes, and longer stays in the hospital, compared with community-acquired peritonitis, which may explain its correlation with the development of VAP [2].

The onset of VAP was late in the majority of patients. More than 90 % of cases of VAP occur within the first 10 days of the intubation [30]. VAP was also developed about 16.8 days after the initiation of the peritonitis. A high percentage of the microorganisms responsible of VAP were multidrug resistant (62.5 %). Regarding the antibiogram, almost half of patients (55.0 %) received an adequate treatment. The low number of patients receiving adequate antibiotic treatment may be a consequence of: (1) the late onset of pneumonia (16.8 ± 15.1 days) since the initiation of the peritonitis. At this time, patients had received other antibiotic treatments for the peritonitis. (2) the peritonitis was the primary target of the antibiotic treatment; VAP was not so. It has been demonstrated that patients who receive inadequate empiric antibiotic treatment have longer hospital stays, higher rates of abscesses, and mortality [31]. For this reason, attention should be given to the antibiogram of each respective center, selecting adequate antibiotics taken into account potential multidrug-resistant microorganisms [1]. The high rate of multidrug resistance found in our study may be correlated with the higher proportion of patients presenting late-onset VAP. In general, etiological microorganisms responsible for the peritonitis were different than for VAP, which is consistent with previous studies [6].

According to published studies, mortality rates attributable to VAP range between 53 and 75 % [14, 15]. Since clinical outcomes depend on the length of the stay in the ICU, one goal of our study was to investigate differences in in-hospital mortality at different endpoints (30, 30–90, and 90 days). VAP patients showed a significant higher mortality rate and longer hospital stay than non-VAP. The 90-day mortality was 47.5 % of VAP patients, a value slightly lower than previous studies. Similar to the incidence, there is a decreasing tendency in the mortality rate as a result of the implementation of preventive strategies [22, 25]. In our study, the mortality rate was actually quite high, even in the non-VAP cohort, for secondary peritonitis. The recent multicenter STOP-IT trial has reported a mortality rate of 1.2 % for patients with complicated intra-abdominal infection [32]. Although this study cohort did have a much higher rate of septic shock and severe sepsis than that our study, most recent severe sepsis and septic shock studies have reported mortality rates of approximately 25 %. Risk factors associated with in-hospital mortality included VAP and SOFA score, although the univariate analysis revealed both VAP and stay at ICU as significant risk factors associated with mortality. VAP was only significant for 30- to 90-day in-hospital mortality. According to the literature, the development of VAP is associated with the percentage of nonsurvivors and survivors in a rate 2:1. It is interesting to note that Kaplan–Meier curves showed a significant divergence in survival likelihood since approximately the day 40th after admission in the ICU. Among the extensive studies evaluating the risk factors associated with worse outcomes and mortality for secondary peritonitis [31, 33–35], none of them have included VAP in their analyses. One possible reason to omit it might derive from the fact that VAP is intrinsically associated with the stay in the ICU, whatever the underlying condition of the patient. Nevertheless, results of our study highlight the importance of including VAP as a factor involved in the 30- to 90-day in-hospital mortality of patient with secondary peritonitis. Our present study had some limitations. One of them was that the study was performed in a single center. A multicenter study might have strengthen results obtained in the study and have reduced factors intrinsically associated with the center, such as the empiric antibiotic treatment or the spectrum of nosocomial pathogenic microorganisms. Another limitation of the study was the low number of patients developing VAP (40). Although similar to the sample size of the literature, a large cohort of patients might also have strengthen our results and diminish inter-individual differences.

## Conclusion

In light of the impact on morbidity and mortality in the ICU, more attention should be given to the concurrent features among VAP and secondary peritonitis. Additional prospective studies, involving large cohort of patients, are required to corroborate these results.
